# A European survey on treatment of hydrosalpinges in infertile women on behalf of the European Society for Gynaecological Endoscopy (ESGE) Special Interest Group (SIG) on Reproductive Surgery

**Published:** 2020-10-08

**Authors:** A Daniilidis, M Nisolle, P Papandreou, S Gordts, H Krentel, G Pados

**Affiliations:** 2^nd^ Dept. OB-GYN, ‘Hippokratio’ General Hospital, Aristotle University of Thessaloniki, Greece; OB-GYN clinic, Citadelle hospital. Liege, Belgium; Life Expert Centre, Leuven, Belgium; OB-GYN clinic, Bethesda Hospital, Duisburg, Germany; 1^st^ Dept. OB-GYN, “Papageorgiou” General Hospital, Aristotle University of Thessaloniki and Centre for Endoscopic Surgery “Diavalkaniko” Hospital, Thessaloniki, Greece

**Keywords:** tubal surgery, laparoscopy, tubal factor infertility, assisted reproductive technology, hydrosalpinx

## Abstract

A questionnaire-based survey was conducted among members of the European Society for Gynaecological Endoscopy (ESGE), with the aim of increasing awareness of the diagnosis and surgical treatment of tubal disease as an alternative to in-vitro fertiliszation (IVF). Seventeen participants (34%) occasionally used a test for prediction of the ovarian reserve before surgery, and the most commonly used test was anti-mullerian hormone assay (39/50; (80%). Laparoscopy was the preferred method for staging tubal disease (43/50; 86%).Thirty-seven (76%) participants always performed salpingectomy or tubal occlusion before the first IVF attempt. Thirty (60%) of the gynaecological surgeons considered the outcome with tubal surgery and IVF to be similar in mild tubal disease, whereas for severe disease, 31/50 (62%) felt that surgery had worse outcome. Among other factors to be considered in choosing a strategy for treating infertility, 20/50 (40%) of respondents listed the stage of disease. The findings of this survey suggest that first-line treatment for women younger than 35 years old with minor tubal pathology, is tubal surgery. IVF appears to be offered if there are other infertility factors, if the patient is >38 years old and if moderate to severe tubal disease is present.

## Introduction

Tubal disease is responsible for 30-40% of cases of female infertility. Pathology of the fallopian tubes may vary from peritubal adhesions and distorted tubal anatomy or damaged fimbriae to hydrosalpinx or tubal blockage (Coughlan and Li, 2011). Pelvic inflammatory disease accounts for more than 50% of tubal disease and is likely to lead to formation of hydrosalpinx (Daniilidis et al., 2017). The risk of infertility is about 8-12% after an episode of pelvic inflammatory disease and it doubles with each subsequent episode (Coughlan and Li, 2011). The most common pathogen associated with tubal disease is Chlamydia trachomatis (Harbet al., 2019). Other reasons for tubal-factor infertility are endometriosis, a history of ectopic pregnancy and previous pelvic surgery (American Society of Reproductive Medicine, 2015). The incidence of hydrosalpinx in infertile women is 30%.The presence of hydrosalpinx is associated with lower pregnancy and live birth rates during an in-vitro fertilisation cycle. In addition, it seems to double the risk of biochemical pregnancy loss and triple the risk of ectopic pregnancy (Harbet al., 2019). A number of methods has been used for the diagnosis of tubal disease in infertile women, e.g., laparoscopy with dye test and transvaginal hydrolaparoscopy (fertiloscopy), which permit the direct visualisation of the pelvic organs and evaluation of tubal patency, and hysterosalpingography, which prevails as a diagnostic method for women without co-morbidities (such as pelvic inflammation or endometriosis) as it is less invasive(Gordts et al., 1998; National Institute for Clinical Excellence and Health, 2013; American Society of Reproductive Medicine, 2015; Gebeh and Metwally, 2017).For the treatment of women with tubal factor infertility, assisted reproductive technology and endoscopic surgical approaches have improved in recent years (Coughlan and Li, 2011; Daniilidis et al., 2017). In vitro fertilisation (IVF) is associated with good per-cycle success rates and it is a less invasive method. The main disadvantages of IVF are the cost (especially if more than one cycle is required); the need for monitoring repeatedly for several weeks; the risks of multiple pregnancy and ovarian hyperstimulation syndrome; and a higher incidence of adverse perinatal outcomes in singleton infants(Daniilidis et al., 2017).Tubal surgery for the treatment of infertility also has advantages; it is usually a minimally invasive, one-time procedure that allows patients to attempt conception spontaneously without further interventions, and multiple attempts may be made (Daniilidis et al., 2017). On the other hand, surgical treatment for tubal disease is associated with an increased risk of surgical complications, such as bleeding, infection, organ damage, and reaction to anaesthesia; postoperative discomfort during the short recovery phase; and higher risk of ectopic pregnancy (Daniilidis et al., 2017). Surgical techniques used for the treatment of tubal disease include salpingectomy, salpingostomy, tubal anastomosis and tubal occlusion (American Society of Reproductive Medicine, 2015).This questionnaire- based survey of members of the European Society for Gynaecological Endoscopy (ESGE) aimed to explore the currently applied practices among gynaecological surgeons with expertise in laparoscopic treatment of tubal-factor infertility. Participants of the survey were not specifically infertility specialists.

## Materials and methods

A questionnaire survey was conducted to assess the approaches of members of the European Society for Gynaecological Endoscopy (ESGE). From March 1^st^ to March 31^st^ , 2019, ESGE members were invited by the ESGE Central Office to complete a 22-item online questionnaire, accessible through the ESGE website. For some questions, more than one answer was acceptable. The invited gynaecological surgeons were not specifically infertility specialists, but they had experience in laparoscopic treatment of tubal-factor infertility. The participants received three reminders during this period. Fifty 50 gynaecological surgeons participated in the survey.

## Results

Forty-eight (96%) of the participants were specialists and two (4%) were trainees. Sixty per cent of the participants were aged 35 to 54 years; 25(50%) were experts who performed more than 90 laparoscopic procedures per year; and 19 (38%) had over 16 years of practice in endoscopic operations. Eighteen (36%) of the participants performed more than 30 laparoscopic tubal operations per year and 22 (44%) performed 10 to 30 per year. Seventeen of the participants (34%) worked in a university teaching hospital and 25 (50%) worked in a national health service hospital.

According to 43 (86%) of the surveyed gynaecological surgeons, the best method for staging tubal disease was laparoscopy. Salpingectomy was the operation preferred by 30 (60%) participants, salpingoneostomy by nine (18%), tubal anastomosis by six (12%) and tubal occlusion by two (4%). Twenty-nine (58%) of the participants preferred bipolar energy for electrocoagulation during surgical treatment for tubal disease. When there is an incidental finding of hydrosalpinx 21(42%) of the gynaecological surgeons reported treatment in all cases; 18 (36%) reported treatment depending on the case; six (12%) reported no treatment; and 4 (8%) reported treatment only in case of bilateral disease. When hydrosalpinx was an incidental finding, 43 respondents (86%) preferred to inform the patient first and perform second-look surgical treatment only when there is indication, such as pain or infertility.

In treatment of asymptomatic hydrosalpinx 23 (46%) gynaecological surgeons reported using no routine antibiotic treatment, 18 (36%) reported using occasional antibiotic treatment; and five (10%) reported using antibiotic treatment in all cases. When antibiotics were used for this condition, 20(40%) of the gynaecological surgeons seemed to prefer a combination of doxycycline, cephalosporin and penicillin, whilst 25 (50%) seemed to prefer doxycycline or cephalosporin only.

Twenty-two (44%) participants reported using a test for predicting ovarian reserve in all cases before tubal surgery for infertility, and 40 (80%) felt the most precise and reliable test for predicting ovarian reserve is the serum anti-Mullerian hormone assay. Twenty-four (48%) reported salpingectomy as the best method for surgical treatment of hydrosalpinx in case of infertility, while 17 (34%) prefer laparoscopic tubal occlusion, six (12%)prefer salpingostomy, and three (6%) prefer hysteroscopic tubal occlusion, with permanent micro inserts to cause fibrosis and irreversible tubal occlusion, which develop within three months of insertion.

Thirty-seven (76%) participants always performed surgical treatment of hydrosalpinx before the first IVF attempt, and 40 (80%) supported salpingectomy as being the best operative method regarding the outcome of a following IVF attempt. For women with mild tubal disease, good ovarian reserve and no other infertility factors, the prognosis of tubal surgery versus IVF is similar according to 30(60%) respondents. For women with moderate tubal disease 19(38%) participants felt that surgery and IVF had similar outcomes, and 17 (34%) felt that surgery had worse outcomes. In the treatment of severe tubal disease, 31 (62%) gynaecological surgeons stated that tubal surgery has worse outcomes than IVF. Twenty-two (44%) of participants reported that the combination of both methods imposes higher cost. Finally, according to 20 (40%) of the surveyed gynaecological surgeons, the stage of tubal disease is the first factor to take into account when considering strategies for treating infertility, whereas 30 (60%) felt that age and other infertility factors are most important.

## Discussion

The results of this survey of 50 members of the ESGE on surgical treatment of tubal-factor infertility are comparable to the published literature on this subject. Thirty-eight (76%) participants favoured laparoscopy as the ideal method for the evaluation of tubal patency and the diagnosis of tubal disease, which is in line with reported practice (Coughlan and Li, 2011;Gebeh and Metwally, 2017). Almost two-thirds of the participants perform laparoscopic salpingectomy or tubal occlusion, which is recommended in cases of tubal disease that may have a detrimental effect on the fallopian tube patency and on in vitro fertilization outcome (American Society of Reproductive Medicine, 2015).The use of bipolar diathermy seems to be preferable to monopolar diathermy according to the surgeons participating in this survey, which is in line with a report of bipolar diathermy being the safer method (Saridogan et al., 2017). Almost half the participants reported using no antibiotics in the treatment of asymptomatic hydrosalpinx, and only 10% reported using antibiotic treatment in all cases. This response corresponds with a report that tubal damage and infertility persist in 8-12% of chlamydia cases even after successful antibiotic treatment (Coughlan and Li, 2011). Twenty-two (44%) participants use a test for predicting the ovarian reserve in all cases before tubal surgery for infertility. That is in line with modern literature, which reports that surgery for tubal disease may be associated with damage to ovarian blood flow and ovarian reserve (Daniilidis et al., 2017).The presence of hydrosalpinx in women undergoing an IVF cycle is associated with lower implantation and pregnancy rates according to retrospective studies (Dechaudet al., 1998;Strandellet al., 1999; Kontoravdiset al., 2006; Minghui and Lin, 2016) and laparoscopic salpingectomy reportedly improves IVF pregnancy rates (American Society of Reproductive Medicine, 2015; Zhang et al., 2015). Participants in this survey seemed to concur with this practice, as (76%) reported that they always treat hydrosalpinx before the first IVF attempt and 80% supported salpingectomy as the best surgical method for this indication. For women with mild tubal disease, a good ovarian reserve and no other infertility factors the prognosis of tubal surgery versus IVF is similar according to 60% of the survey answers. This opinion perhaps does not fully comply with literature, as it is stated that in case of mild tubal disease, (limited filmy adhesions, mildly dilated tubes, thin and pliable walls and lush endosalpinx with preservation of the mucosal folds) reconstructive tubal surgery seems to be effective, with a rate of spontaneous intrauterine pregnancy of 58–77%. On the other hand, for severe tubal disease, 62% of the gynaecological surgeons stated that tubal surgery is associated with worse prognosis than with IVF. This response is in line with evidence in modern literature, that surgical treatment of severe tubal disease (presence of extensive, dense peritubal adhesions; massively dilated tubes; thick, fibrotic walls; and sparse or absent luminal mucosa) has a poor prognosis, with a rate of intrauterine of 0–22% (Balen and Rutherford, 2007; Sacks and Trew, 2004; National Institute for Health and Clinical Excellence, 2013; Gomel, 2015).

## Conclusions

Based on the survey responses, first-line treatment for women less than 35 years old with minor tubal pathology seems to be tubal surgery. IVF appears to be offered when there are other infertility factors, if the patient is >38 years old, and when moderate- to-severe tubal disease is present. According to the surveyed laparoscopic surgeons, tubal surgery and assisted reproductive technology in most of the cases are not competitive techniques, but usually tubal surgery is a prerequisite for the success of the IVF. Optimizing pregnancy rates and reducing the risks associated with IVF or surgical treatment should always be the main goal. The outcome of our survey is especially meaningful because of the high level of expertise in laparoscopic surgery and the high level of expertise in the laparoscopic treatment of hydrosalpinx among the participating gynaecological surgeons. On the other hand, the insufficient number of participants is a drawback, which points out the need for a future survey, better designed and with more gynaecologists with experience in laparoscopic treatment of tubal-factor infertility. Such a survey would minimize bias and produce clear answers and recommendations.
